# Observation and motor imagery balance tasks evaluation: An fNIRS feasibility study

**DOI:** 10.1371/journal.pone.0265898

**Published:** 2022-03-23

**Authors:** Latifah Almulla, Ibraheem Al-Naib, Ijlal Shahrukh Ateeq, Murad Althobaiti

**Affiliations:** Biomedical Engineering Department, College of Engineering, Imam Abdulrahman Bin Faisal University, Dammam, Saudi Arabia; University rehabilitation institute, SLOVENIA

## Abstract

In this study, we aimed at exploring the feasibility of functional near-infrared spectroscopy (fNIRS) for studying the observation and/or motor imagination of various postural tasks. Thirteen healthy adult subjects followed five trials of static and dynamic standing balance tasks, throughout three different experimental setups of action observation (AO), a combination of action observation and motor imagery (AO+MI), and motor imagery (MI). During static and dynamic standing tasks, both the AO+MI and MI experiments revealed that many channels in prefrontal or motor regions are significantly activated while the AO experiment showed almost no significant increase in activations in most of the channels. The contrast between static and dynamic standing tasks showed that with more demanding balance tasks, relative higher activation patterns were observed, particularly during AO and in AO+MI experiments in the frontopolar area. Moreover, the AO+MI experiment revealed a significant difference in premotor and supplementary motor cortices that are related to balance control. Furthermore, it has been observed that the AO+MI experiment induced relatively higher activation patterns in comparison to AO or MI alone. Remarkably, the results of this work match its counterpart from previous functional magnetic resonance imaging studies. Therefore, they may pave the way for using the fNIRS as a diagnostic tool for evaluating the performance of the non-physical balance training during the rehabilitation period of temporally immobilized patients.

## Introduction

Physical training on balance tasks has shown to be an effective approach for young and elderly subjects to enhance their postural control and decrease the falling risks [1, 2]. However, patients suffering from immobilization due to some injuries or diseases are not able to perform this kind of training. The rate of falling, loss of mobility, and mortality risk dramatically increase for people with long-term immobilization [3]. Thus, several studies have suggested an alternative, non-physical balance training to reduce postural control loss after the immobility period, such as action observation (AO) or motor imagery (MI) [4, 5]. Training by AO has shown to be an effective method for enhancing the performance of standing, sitting, and walking activities in elderly participants and chronic stroke patients [6–8]. Also, MI training was demonstrated to enhance balance and gait abilities within older adults and post-stroke patients [9–11]. Moreover, a combination of AO and MI (AO+MI) training of balance tasks has been shown to enhance postural control on highly variable and unpredictable balance movements with healthy participants that followed an actor performing balance task [12]. The enhancement of physical task performance after the AO experiment is perhaps due to the overlap between the activated brain regions during the actual motor execution and the AO. This also has been observed during actual motor execution and MI [13, 14]. Moreover, an accepted hypothesis states that the motor system is activated during multiple conditions that are related to either self-intended actions or observed actions from another person [15]. Therefore, from these studies, it is concluded that AO, MI, and AO+MI training tasks have a positive effect in enhancing postural control.

Positron emission tomography (PET) was utilized to study brain activation during MI of static balance task. This task induces activation in the dorsal premotor area bilaterally, left dorsolateral prefrontal cortex, left inferior parietal lobule, precuneus bilaterally, and right posterior cingulate cortex [16]. Taub *et al*., utilized functional magnetic resonance imaging (fMRI) to locate the neural sites related to AO and MI during different postural control tasks [17]. They reported that the AO+MI experiment of dynamic standing balance task evokes activation in the supplementary motor area (SMA), premotor cortex, primary motor cortex, basal ganglia (putamen), and cerebellum. The study also showed that the more challenging balance task such as mediolateral perturbation on an inclined surface evokes a higher activation in the brain in comparison to normal standing. The authors concluded that AO+MI is the best scheme for training on challenging balance tasks in comparison to the balance training tasks by AO or MI only. More recently, another fMRI study reported that elderly subjects have higher brain activation compared to young participants, particularly in the demanding dynamic balance task when following AO + MI training tasks [18]. Despite the evidence from these findings that training by AO, MI, or AO+MI evoked different brain regions, the full understanding and evaluation of the brain activation patterns during these non-physical balance control training tasks have not been fully explored using all available neuroimaging modalities.

Among the neuroimaging modalities, fNIRS has the potential to facilitate the measurements of task-related cortical responses since it has a lower cost and a higher temporal resolution in comparison to PET or fMRI [19]. Also, EEG is limited with spatial resolution because of the volume conduction effect [20, 21]. Thus, many studies considered fNIRS to have a better spatial resolution in comparison to EEG [19, 22, 23]. Furthermore, fNIRS is a portable modality that allows the study of neurocognitive processes in real environments without any restrictions on the subject’s posture and motion. Moreover, it can be integrated with other neuroimaging modalities, such as EEG [24]. Due to these advantages of fNIRS over other neuroimaging modalities, several studies utilized fNIRS modality for not only studying the real execution of motor tasks but also for studying motor imagery and action observation-based tasks [25–27]. fNIRS measures the relative change in hemoglobin concentrations by the means of backscattered near-infrared light from the human brain tissues [28]. Conventionally, fNIRS detects brain activities by utilizing two wavelengths to measure the variations in oxyhemoglobin (HbO) and deoxy-hemoglobin (HbR) concentration [29, 30]. fNIRS has been used in lower limb rehabilitation for investigating the brain activation patterns during standing and sitting [26], walking [31, 32], running [33], precision stepping [34], and many other applications reviewed in Refs. [35–38].

Previous fNIRS studies have shown significant activations in the prefrontal cortex during the actual execution of a board balance task [39–42]. Other groups used fNIRS to illustrate the role of SMA in postural balance control [43, 44]. Moreover, some other studies were carried out and investigated hemodynamic responses measured by fNIRS during the actual execution of balance tasks [37]. However, studying hemodynamic responses using fNIRS during the non-physical balance training has not been yet investigated. More specifically, it is important to have a cost-effective and easy-to-use neuroimaging modality, such as fNIRS, as a diagnostic tool to evaluate the performance and the progress of the non-physical balance training, especially during the rehabilitation period of temporally immobilized patients.

In this work, we aim to investigate the ability of fNIRS to measure the hemodynamic response evoked by AO, AO+MI, and MI of different demanding balance tasks, namely static and dynamic standing, in healthy participants. We expect an activation in the motor areas from the concept of the motor neuron system that states that the motor areas are activated during the observation of a task performed by another person [45]. Furthermore, the prefrontal cortex was previously shown to be active when observing other’s person tasks [46] as well as its important role in motor imagery tasks, and more specifically in the tasks related to gait and lower limb movements [47, 48]. Thus, the motor and prefrontal areas are studied in this work. We hypothesized that: (i) a higher level of activations with the increase of balance task complexity in comparison to a lower demanding balance task; and (ii) a higher level of activations during AO+MI experiment than during AO or MI experiments during dynamic balance task.

## Methods

### Experimental setup

**Fig 1:**
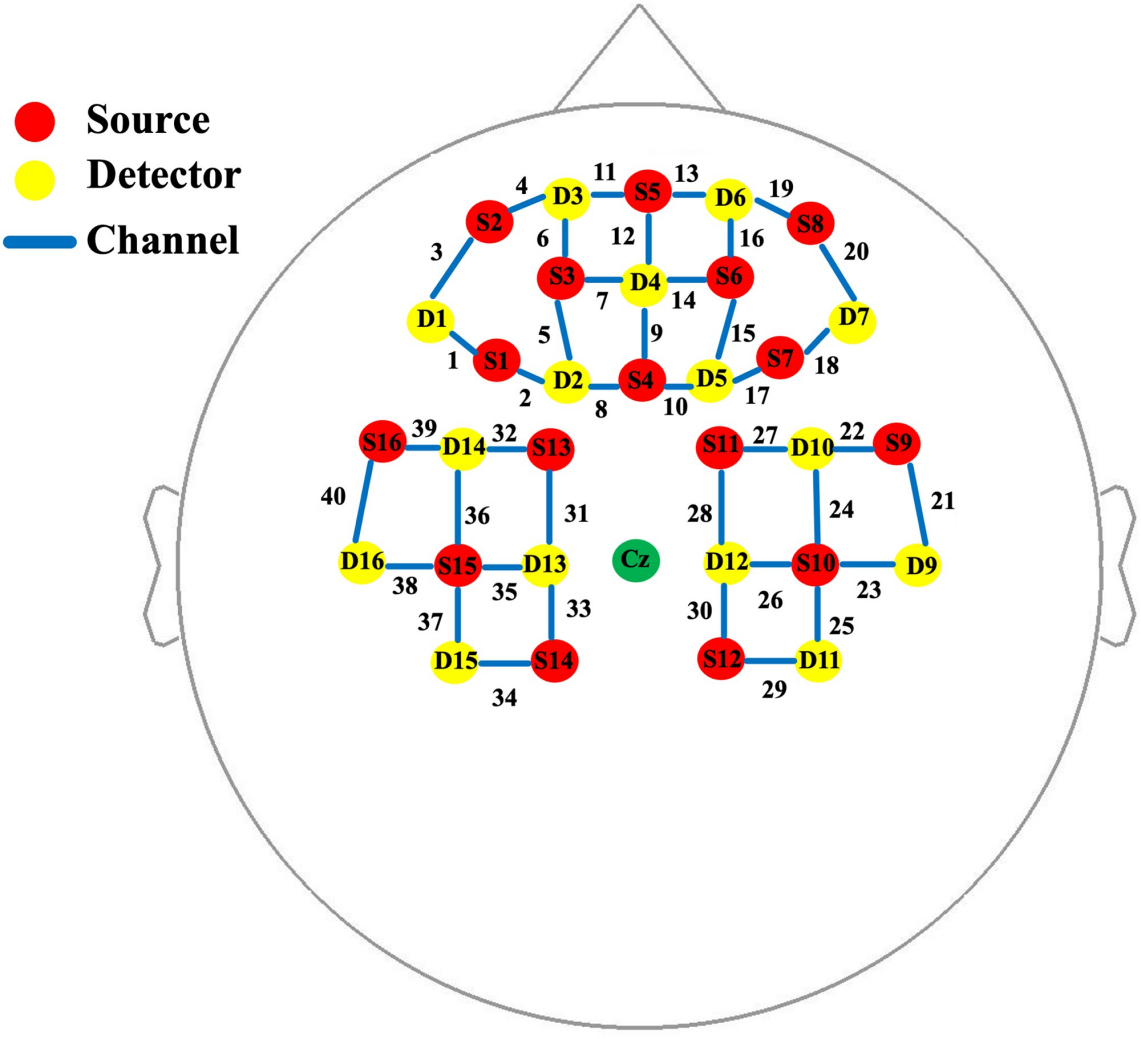
Optodes placement with channel numbers configuration placed on prefrontal and motor regions. Sources are indicated by red, detectors are indicated by yellow, and channels are indicated by blue. Cz point is indicated by green as a reference point.

In this study, we used a continuous wave fNIRS system (tandem NIRSPORT 2 fNIRS system, from NIRx Medical Technologies, LLC) that operate on two wavelengths, 760, and 850 nm. Previous fNIRS studies showed significant hemodynamic activation in prefrontal and motor cortices during the actual execution of balance tasks [39–44]. Hence, sixteen sources and fifteen detectors were placed in the prefrontal cortex as well as in the right and left hemispheres of the motor cortex. As a result, the data from 40 different channels were recorded. The source-detector distance in the experimental setup ranges between 2.9 cm to 3.1 cm with a nominal value of 3.0 cm in most cases. Fig 1 illustrates the source-detector configuration with the channel numbers used for recording the brain activations data. fOLD toolbox was used to find out the position of fNIRS optodes based on the 10–20 EEG coordinates system according to a set of brain regions of interest [49]. Seventeen healthy subjects with no history of any neurological orthopedic or visual disorders were participated in this study (mean age of 32±11, fifteen male subjects, and two female subjects). The acquired data from four subjects showed very low data quality, due to dense dark hair with a variation coefficient higher than 7.5% for most of the channels. Therefore, their data were excluded from the analysis, leaving the data of thirteen subjects for the analysis. Before the experiments, the participants were briefed on the experiments and asked to sign a written informed consent. All the experiments were conducted in accordance with the Institutional Review Board for research ethics at Imam Abdulrahman Bin Faisal University.

### Experimental paradigm, stimuli, and procedure

Prior to the start of the study, each participant was asked to be familiarized with the tasks before the start of data recordings. In this familiarization period, the subjects were asked to watch two videos of balance tasks: static standing and dynamic standing. The static standing balance task video showed a person standing normally with an upright posture and without any movements. The dynamic standing balance task video presented a person balancing a mediolateral perturbation while standing on a balance board.

**Fig 2:**
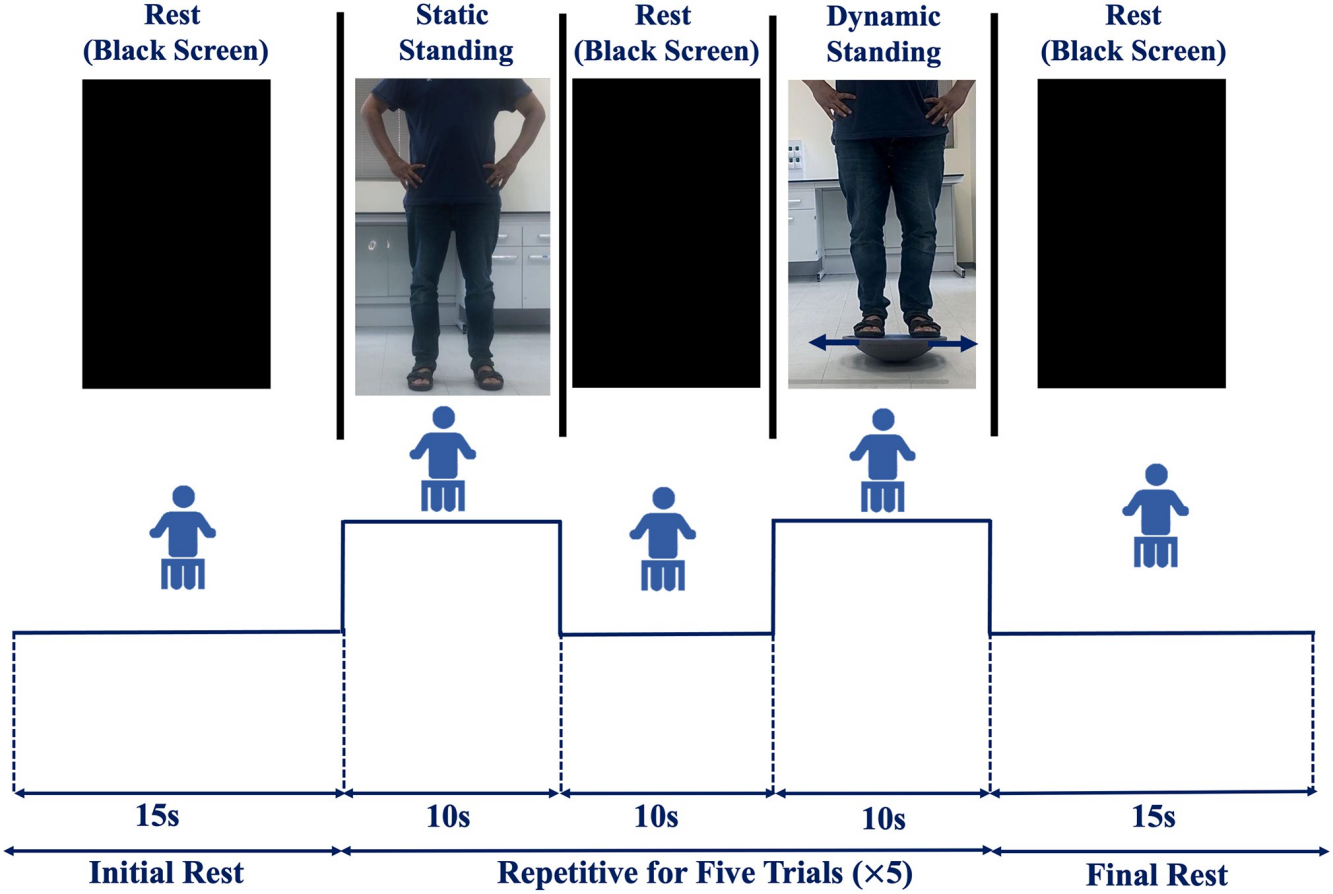
Experimental paradigm of the three (AO, AO+MI, and MI) experiments. Subjects watched two videos: static standing balance task (normal standing) and dynamic standing balance task (balancing a mediolateral perturbation), during two experiments: (AO) and while imagining themselves as the person performing the tasks (AO+MI). During the third experiment, participants verbally instructed, through previously recorded voice instructions, to close their eyes and imagine themselves performing static and dynamic balance tasks. Each subject repeated each task five times with a resting period of 10s between the two balance tasks.

Next, AO, AO+MI, and MI conditions were performed in order, with three minutes break period between each experiment and the other. Fig 2 illustrates the experimental paradigm of the three experiments. The experiments started and ended with a resting period of 15s where the subjects were sitting on a chair during the whole period of the experiments, at a distance of 1.25m from a screen measuring 1.80m×1.20m. The paradigm included static standing and dynamic standing balance tasks repeated five times for each condition. The length of each task was 10s followed by 10s of resting period. The trials were altered alternatively between static standing and dynamic standing balance tasks.

During the AO experiment, the participants were instructed to simply watch the videos of the static and dynamic balance tasks when they presented on the screen and to relax if a black screen appeared. Another session was performed for AO+MI where the subjects were instructed to watch the two videos and imagine themselves as the ones who are performing the static and dynamic balance tasks Moreover, participants were asked to relax when the black screen is presented. Finally, for the MI experiment, the participants were instructed to close their eyes, and follow the instructions from the played audio to imagine themselves doing either the static standing task, dynamic standing task, or to relax. The experimental paradigm procedures were coded and presented by the PsychoPy software platform [50].

### Data analysis

The fNIRS data analysis was computed by NIRS Brain AnalyzIR Toolbox [51] working on MATLAB 2018 (MathWorks, Natick, MA, USA). In the beginning, the optical density signals were calculated from time series data and then the changes in HbO and HbR were calculated using modified Beer-Lambert Law [52] with a differential pathlength factor of 6 for both wavelengths. The subject-level analysis was based on a statistical autoregressively whitened weighted least-squares regression model [53]. In the last few years, this model has been increasingly used for the analysis of fNIRS data in several studies such as in Refs. [54–56]. The algorithm of this model showed better sensitivity-specificity characteristics in comparison to other analysis approaches of the general linear model, such as statistical parametric mapping and the ordinary least-squares model [57]. This model was designed to address the serially correlated noise errors coming with the physiological noise of fNIRS raw data by considering them as statistical outliers. This model iteratively reweights all error terms to minimize the effect of outliers using both pre-whitening and robust regression, thus making the algorithm robust to physiological noise and motion artifacts as well. Therefore, using any pre-processing technique (such as principal component analysis or band-pass filtering) was not explicitly required to remove these components [58–60]. Furthermore, in order to remove the strong noise component presented at very low frequencies, a high pass filter based on a discrete cosine transform, with a 120s cut-off period, was utilized in this study [61, 62]. The conical hemodynamic response function was applied as a basis function to model the hemodynamic response. To generalize the results for all the subjects, the resulting data from the subject-level analysis were submitted to a second, group-level analysis. The analysis of the group level was computed through the linear mixed-effects model. To confirm the absence of physiological noise, HbO and HbR chromophores are visually checked whether they are going in the same direction or not. For each channel, t-tests with different types of contrasts were computed to evaluate the hypotheses of this study. In order to avert Type I error presented in multiple comparisons, false discovery rate (FDR) correction was applied [63, 64]. The threshold level of significance was set to p < 0.05 (FDR corrected). The signal processing steps performed in this study were checked to be aligned with the recommendations for using fNIRS in balance and gait research proposed in Ref. [65].

To calculate the T-score for each channel independently, the contrast tests were performed for HbO data. We concentrated in this study on HbO data rather than HbR data. That is because the HbR signals have lower amplitude levels and accordingly a lower signal-to-noise ratio in comparison to HbO signals. In this study, the first contrast was computed to evaluate the significantly activated channels during AO, AO+MI, and MI experiments against the baseline for both static and standing balance tasks (contrast: task > baseline). A contrast between static and dynamic standing tasks was conducted to evaluate the effect of the complexity of the balance task on the level of HbO (contrast: static standing < dynamic standing). Finally, a comparison between AO+MI and the other two experiments (contrasts: AO experiment < AO+MI experiment and AO+MI experiment > MI experiment) was conducted.

## Results

### Hemodynamic responses for static and dynamic standing balance tasks

**Fig 3:**
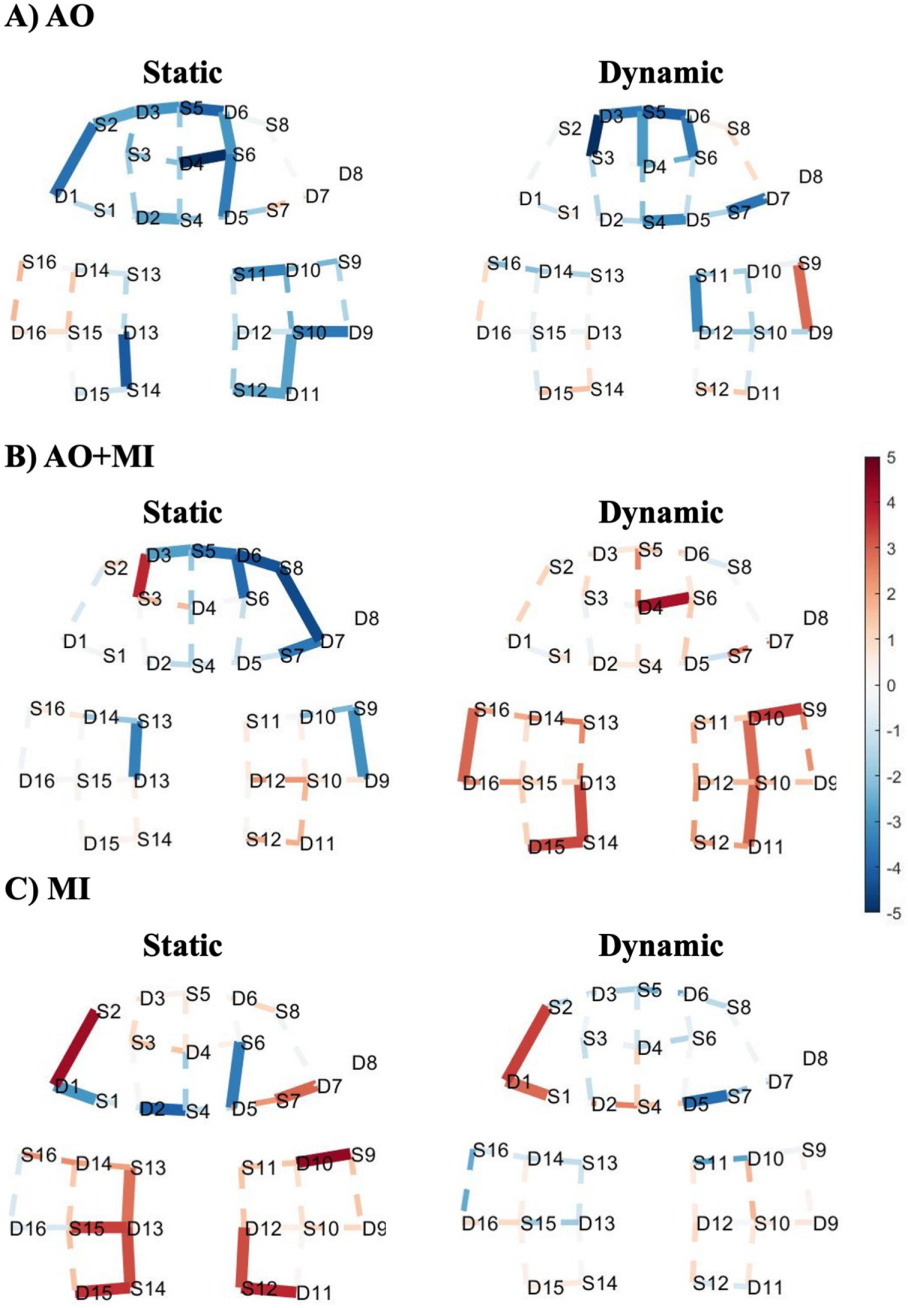
T-map of oxy-hemodynamic response (HbO) corresponding to (A) action observation experiment, (B) a combination of motor imagery and action observation experiment, and (C) motor imagery experiment during static standing balance task (left) and dynamic standing balance task (right). These maps are generated by contrasting each task against the baseline. Significantly activated channels at p < 0.05 (FDR corrected) are indicated by thick and solid lines. The red colour indicates stronger task activity against the baseline. The dashed line shows the channels that were not statistically significant. This map was generated by using NIRS Brain AnalyzIR Toolbox [51]. Fig 2 illustrates the referencing for the brain regions with 10–20 EEG system positions.

**Fig 4:**
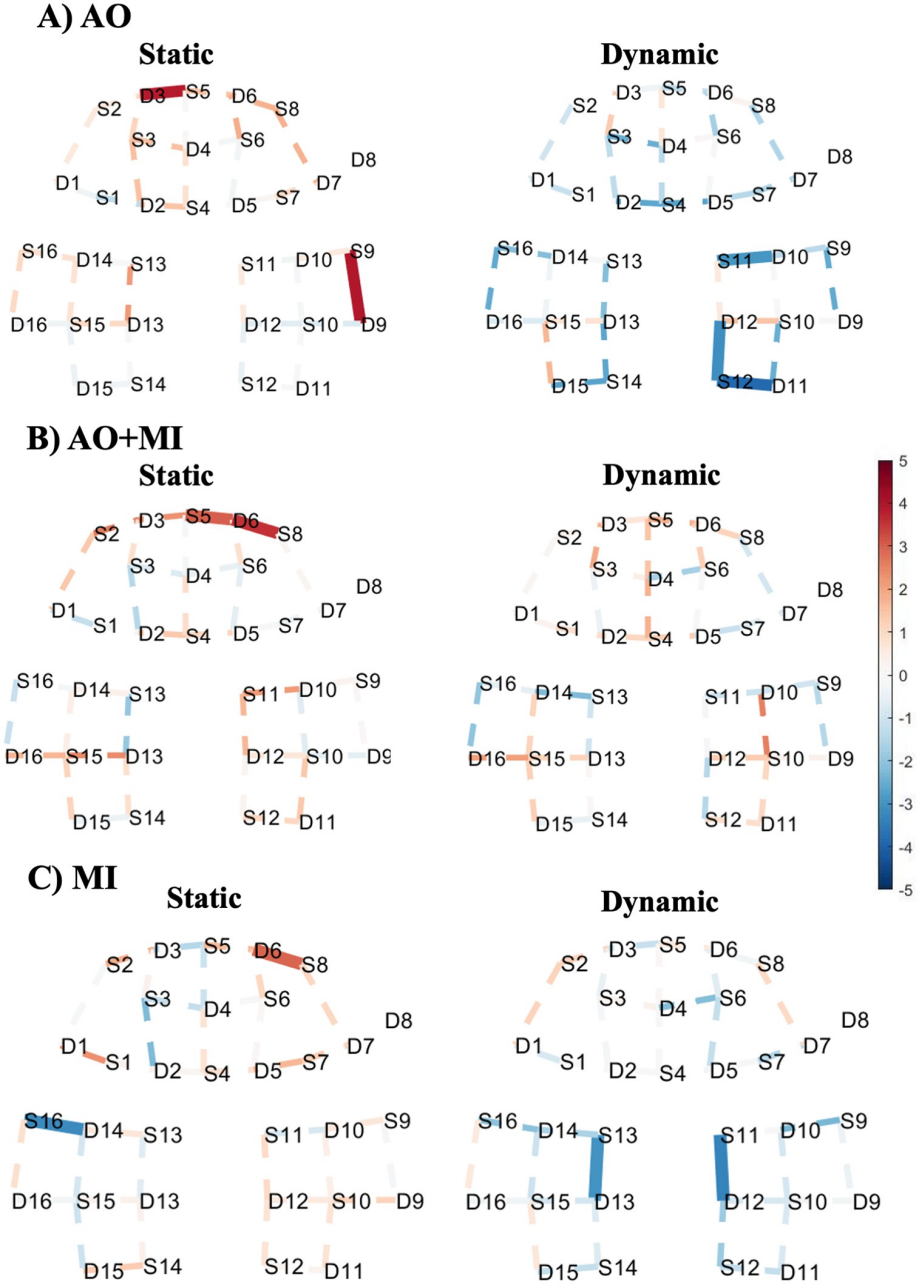
T-map of deoxy-hemodynamic response (HbR) corresponding to (A) action observation experiment, (B) a combination of motor imagery and action observation experiment, and (C) motor imagery experiment during static standing balance task (left) and dynamic standing balance task (right). These maps are generated by contrasting each task against the baseline. Significantly activated channels at p < 0.05 (FDR corrected) are indicated by thick and solid lines. The red colour indicates stronger task activity against the baseline. The dashed line shows the channels that were not statistically significant. This map was generated by using NIRS Brain AnalyzIR Toolbox [51]. Fig 2 illustrates the referencing for the brain regions with 10–20 EEG system positions.

T-map of the changes in the HbO responses shown in Fig 3, depicts the activation patterns related to each experimental task in comparison to the baseline. For the results of the AO experiment shown in Fig 3(A), no significant increase in channels is observed in the static standing condition. On the other hand, the dynamic standing condition revealed a significant relative increase in activation in the right motor area. As shown in Fig 3(B), more significantly activated channels are observed during the AO+MI experiment for both static and dynamic balance tasks. The static condition showed significant activation in the prefrontal area, while the dynamic condition induced activation in the prefrontal area, and both right and left motor regions. Fig 3(C) demonstrates the MI experiment activation of the static standing task showed significant activation in the prefrontal region and concentrated more with a relatively higher number of significant channels in the motor area. For the dynamic standing task, significant channels are observed in the prefrontal area. Furthermore, HbR data for AO, AO+MI, and MI during static and dynamic standing tasks against the baseline is shown in Fig 4. Comparing the results presented in Fig 4 and the results shown in Fig 3, a confirmation for the absence of any physiological noise contamination is validated through visually observing the opposite trend of the HbO compared to HbR data for most of the channels. For instance, negatively correlated channels that are observed visually are channel 13, channel 19, and channel 1 in the static condition of AO, AO+MI, and MI experiments, respectively. On the other hand, other channels that are not negatively correlated were observed as well, for instance, channel 8 during the static condition of the AO+MI experiment. Nevertheless, all the observed positively correlated channels are not statically significant. Thus, the overall results and the conclusion are not affected due to this observation.

### Comparison between static and dynamic standing balance tasks

**Fig 5:**
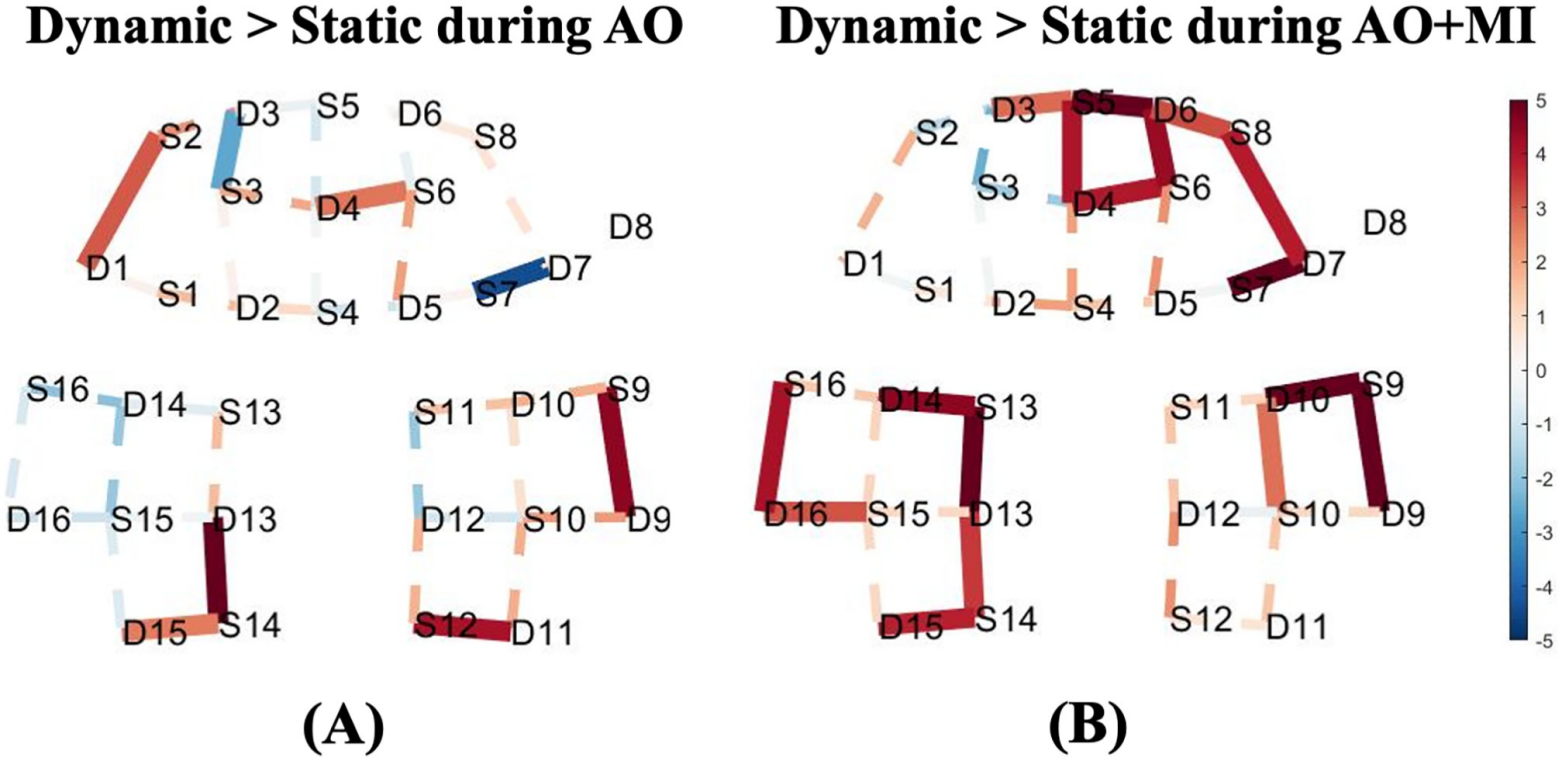
T-map of oxy-hemodynamic response (HbO) corresponding to the contrast between the dynamic standing and static standing balance tasks for (A) action observation experiment, and (B) a combination of motor imagery and action observation experiment. Significantly activated channels at p < 0.05 (FDR corrected) are indicated by thick, solid lines, red colour indicates stronger activity from the dynamic standing task than the static standing task. The dashed line shows the channels that were not statistically significant. This map was generated by using NIRS Brain AnalyzIR Toolbox [51]. Fig 2 illustrates the referencing for the brain regions with 10–20 EEG system positions.

A dynamic standing balance task was contrasted against the static standing balance task to examine the effect of balance task complexity on the level of HbO. For the AO experiment, this contrast showed significant activities in the prefrontal and the motor areas as shown in Fig 5(A). During the AO+MI experiment, more extended cortical areas involving most of the channels in prefrontal and motor cortices revealed a relatively higher activity for this comparison as shown in Fig 5(B). The statistically significant channels are located in the prefrontal cortex, with more concentration in the right region, and bilaterally in the motor cortex. Similarly, during the MI experiment, a relatively higher activation for the dynamic standing in comparison to static standing is observed in the prefrontal region only.

### Comparisons of AO+MI experiment against AO and MI experiments

**Fig 6:**
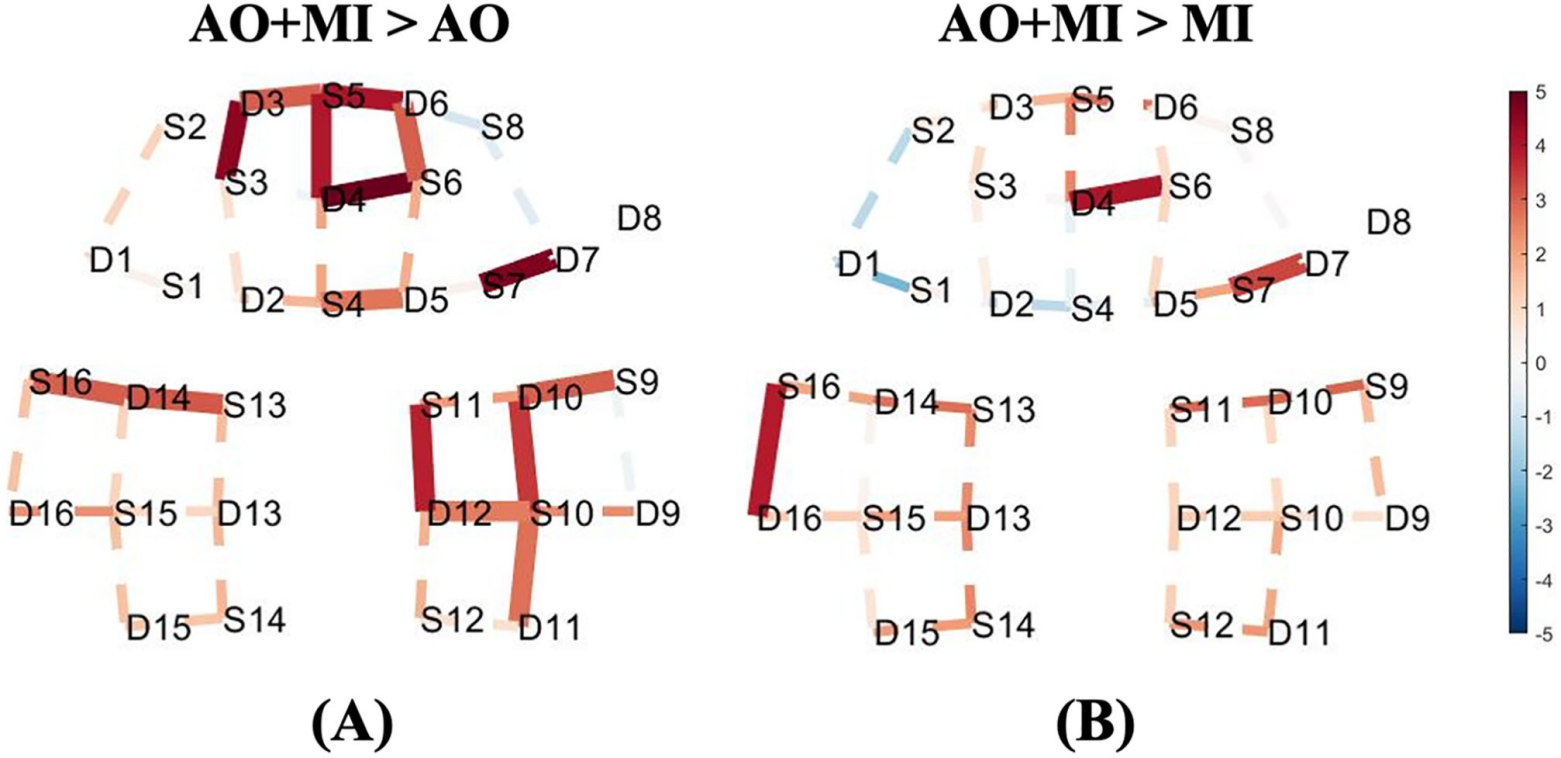
T-map of oxy-hemodynamic response (HbO) corresponding to the contrast between (A) AO + MI and AO experiments, (B) AO + MI and MI experiments of the dynamic standing task. Significantly activated channels at p < 0.05 (FDR corrected) are indicated by thick, solid lines, red colour indicates stronger activity from AO + MI experiment than the AO experiment in **(A)** and MI experiment in **(B)**. The dashed line shows the channels that were not statistically significant. This map was generated by using NIRS Brain AnalyzIR Toolbox [51]. Fig 2 illustrates the referencing for the brain regions with 10–20 EEG system positions.

The contrasts between the AO+MI experiment and the other two experiments were computed to show the effect of combining AO and MI on the changes of HbO level. Furthermore, this contrast was conducted to help in recommending the best experiment that can give a better response and accordingly can be used during the rehabilitation period of temporally immobilized patients. Fig 6(A) shows a comparison between the HbO changes in AO+MI and AO experiments (contrast: AO+MI > AO) during the dynamic standing balance task. This contrast showed relatively higher significant activities for AO+MI in many channels in the prefrontal and motor regions. To validate the preference of AO+MI over other experiments, the AO+MI experiment was also contrasted against the MI experiment (contrast: AO+MI > MI) during the dynamic standing balance task. As illustrated in Fig 6(B), the AO+MI experiment induced relatively higher activities in most of the channels. Only three statistically significant channels are observed in the right prefrontal cortex and left motor cortex.

## Discussion

In this study, we examined the feasibility of using fNIRS to measure the hemodynamic responses in prefrontal and motor cortices for three different types of subject engagements (AO, AO+MI, and MI) during different postural tasks. In each of the three designed experiments, the subjects were asked to follow both static and dynamic standing balance tasks. As shown in Fig 3(A), there is no significant increase in the activations of HbO levels during the AO experiment with respect to the baseline. Interestingly, the results show deactivation patterns in HbO levels with respect to the baseline at some channels in both static and dynamic standing tasks. This observation about the inverse oxygenation response was found at both individual and group levels data in which the HbO response relative decrease and HbR response relative increase. Indeed, this phenomenon of inverse oxygenation was found in some of the previous fNIRS studies for MI experiments [66, 67]. Inadvertent participant movement during resting periods was shown as an explanation for this phenomenon [68]. This reason might be also reflected in AO experiment data. Another possible reason is the usage of the resting period as a control condition, in which participants might evoke another type of activates, like planning, instead of just resting. Nevertheless, further investigations regarding this point are needed to find out if the AO tasks always show deactivation patterns.

For the AO+MI and MI experiments, there are many statistically significant activated channels in prefrontal or motor regions of both static and dynamic standing balance tasks as presented in Fig 3(B) and 3(C). More specifically, channels 6 and 14 are relatively activated in the frontopolar area for static and dynamic standing tasks during the AO+MI experiment, respectively. Moreover, the dynamic standing balance task of AO+MI activates the premotor and supplementary motor cortex (channel 24). These areas are also activated during the static standing balance task of the MI experiment (channel 31). However, no activations are indicated in the frontopolar area during the MI experiment, which perhaps is due to the absence of visual input. The premotor and supplementary motor areas are known to be important for the actual execution of postural control [17, 18]. Hence, the fNIRS systems might be a potential neuroimaging modality to track the non-physical balance training from prefrontal and motor cortices. Specifically, in premotor and supplementary motor cortices during AO+MI and MI experiments. As fNIRS systems are quite compact and offer reliable information in this regard, it has a huge potential over the fMRI.

For most of the channels shown in Fig 5, we found that the increase of balance task complexity results in a greater significant hemodynamic response (HbO) level in comparison to less demanding balance tasks during AO and AO+MI experiments This finding agrees with a recent study that showed an increase in prefrontal cortical activity with progressively more difficult actual balance behaviors [69]. The statistically significant differences between static and dynamic balance tasks are in the frontopolar area for both AO (channel 14) and AO+MI (channel 11 to channel 14 and channel 16) experiments. Moreover, the AO+MI experiment showed a significant difference between the two tasks in areas that are thought to be related to balance control, which are premotor and supplementary motor cortices (channel 31). Previous fMRI studies showed no significant activation between dynamic and static standing balance tasks during the MI experiment [17, 18]. Interestingly, this study shows that the static standing task induces higher activation patterns in comparison to the dynamic standing task on the motor region during the MI experiment. This finding was unexpected, which might indicate that the participants used an alternative strategy instead of the exact simulation of static or dynamic balance tasks during the imagination. Furthermore, this also may result from the motor imagery abilities differences between one participant and another.

The results of the contrast between the AO+MI experiment and the other two experiments show that the AO+MI experiment evoked higher HbO responses in comparison to AO or MI alone (Fig 6). Thus, suggesting the AO+MI experiment as the best experiment that can be used during the rehabilitation period of temporally immobilized patients. A possible reason for this finding is that combining AO with MI might enable the participants to have a much better kinaesthetic experience and physiological sensations of the followed balance tasks. Remarkably, the results of this study are in line with those findings from an earlier fMRI study [17]. Furthermore, the results of contrasting static and dynamic standing balance tasks tie well with a previous fMRI study [18]. Nevertheless, the fNIRS technique has many advantages over the fMRI including robustness to noise, and portability that allows the measurements to be carried out in a much more realistic environment without any restrictions on the subject’s movements. Furthermore, it has a much higher temporal resolution, lower cost, and is capable of integrating with other neuroimaging modalities.

In this study, due to the lengthy tasks that the participants had to perform, active balancing experiments were not conducted. The participants were rather exhausted after performing the steps of the three different types of experiments. In the future, comparing the results of this study and the active balancing condition could be conducted to show the similarity of the obtained signals with the AO+MI condition only, to reduce the overall experimental paradigm time. Another possible limitation of this study is the use of the resting period as a control condition. A resting state might evoke brain activation related to the subject thought, for instance, planning, thinking of others, or sleepiness. Therefore, comparing these activities with the AO condition showed a relative decrease in brain activation. Future studies could be conducted with another type of criterion for the baseline such as showing someone sitting instead of resting. Moreover, randomization of experiments order was not considered in the study to be consistent with the pervious fMRI study [17], to enable the comparison between fNIRS and fMRI modalities. Finally, the current work could be extended to study the influence of AO, AO+MI, and MI in enhancing the postural control of mobilized participants with female and male participants.

## Conclusion

In conclusion, we have examined in this study the prospect of fNIRS to be utilized for studying static and dynamic standing balance tasks through AO, AO+MI, and MI experiments. In the rehabilitation period of temporally immobilized patients, it is quite essential to assess the performance and the progress of the non-physical balance training through an easy-to-use and cost-effective neuroimaging modality such as fNIRS. We found that many channels in prefrontal or motor regions are significantly activated during the AO+MI experiment of static and dynamic standing balance tasks. These activations are also persistent in the MI experiment. Furthermore, the AO experiment showed almost no significant increase in activations in most of the channels for both balance tasks. Contrasting between static and dynamic balance tasks revealed higher activation patterns during the more demanding task, specifically during AO and AO+MI experiments. Moreover, we showed that AO+MI activated greater HbO responses in comparison to having MI or AO alone. The results of this study may pave the way for fNIRS to become an alternative modality over fMRI in this regard. Furthermore, these findings suggest that fNIRS can perhaps be utilized as a diagnostic tool for evaluating the performance of the non-physical balance training during the rehabilitation period of temporally immobilized patients. Further experiments could be carried out on temporally immobilized patients to verify the potential feasibility of utilizing fNIRS as a diagnostic tool. This was a preliminary study that aimed at showing the feasibility of using fNIRS in measuring the hemodynamic response evoked by AO, AO+MI, and MI of different demanding balance tasks. In the future, it would be useful to include non-healthy subjects to shed more light on the feasibility of using fNIRS in this regard.
